# Gramine promotes functional recovery after spinal cord injury via ameliorating microglia activation

**DOI:** 10.1111/jcmm.16728

**Published:** 2021-07-19

**Authors:** Xiaolang Lu, Fengfeng Lu, Jiachen Yu, Xinghe Xue, Hongyi Jiang, Liting Jiang, Yang Yang

**Affiliations:** ^1^ Department of Orthopedics The Second Affiliated Hospital and Yuying Children’s Hospital of Wenzhou Medical University Wenzhou China; ^2^ The Second School of Medicine Wenzhou Medical University Wenzhou China; ^3^ Zhejiang Provincial Key Laboratory of Orthopedics Wenzhou China

**Keywords:** Anti‐inflammation, Gramine, Microglia, NF‐κB pathway, Spinal cord injury

## Abstract

In recent years, a large number of studies have reported that neuroinflammation aggravates the occurrence of secondary injury after spinal cord injury. Gramine (GM), a natural indole alkaloid, possesses various pharmacological properties; however, the anti‐inflammation property remains unclear. In our study, Gramine was investigated in vitro and in vivo to explore the neuroprotection effects. In vitro experiment, our results suggest that Gramine treatment can inhibit release of pro‐inflammatory mediators. Moreover, Gramine prevented apoptosis of PC12 cells which was caused by activated HAPI microglia, and the inflammatory secretion ability of microglia was inhibited by Gramine through NF‐κB pathway. The in vivo experiment is that 80 mg/kg Gramine was injected orthotopically to rats after spinal cord injury (SCI). Behavioural and histological analyses demonstrated that Gramine treatment may alleviate microglia activation and then boost recovery of motor function after SCI. Overall, our research has demonstrated that Gramine exerts suppressed microglia activation and promotes motor functional recovery after SCI through NF‐κB pathway, which may put forward the prospect of clinical treatment of inflammation‐related central nervous diseases.

## 1 INTRODUCTION

The pathogenesis of Spinal cord injury (SCI) comprises the following two stages: primary injury and secondary injury. Primary injury is mainly caused by violence or trauma. After that, secondary injury is initiated; it is including inflammatory response, haemorrhage and ischaemia, hypoxia, demyelination, which induce neuronal death and lead to permanent neurological deficits.^1^, ^2^, ^3^ A series of pro‐inflammatory cytokines, such as IL‐1β, IL‐6, reactive oxygen species (ROS) and nitric oxide (NO) which secreted by activated microglia, would rapidly through the broken blood spinal cord barrier into the injury site.^2^ Although the macrophages and pro‐inflammatory cytokines permeating to the injured site trigger the healing process,^4^ this inflammatory microenvironment results in scar formation, neural apoptosis and axonal demyelination,^5^, ^6^ which consider as a vital pathological factor in functional recovery after SCI.^7^ Thus, the therapy strategy targeting microglia activation and the following inflammatory reaction has been shown to improve the recovery in SCI patients.^8^

Chemically reactive substances extract from dietary plants performed multiple bioactivates, such as anti‐angiogenesis, anti‐inflammation, anti‐proliferation, anti‐apoptosis and chemoprevention effects.^9^, ^10^ Mass of research has demonstrated that the green barley extracts could exhibit excellent pharmacological effects in nervous system and cardiovascular system.^11^, ^12^ The main ingredients from young barley (*Hordeum vulgare L*.) are Gramine, which is indole alkaloid and performed remarkable pharmacological activities including anti‐oxidant, anti‐proliferation and anti‐inflammation reaction.^13^, ^14^, ^15^ However, the anti‐inflammation of Gramine during spinal cord injury and its underlying mechanism are not yet elucidated.

Recent studies have showed that NF‐κB pathway involved in the neuroinflammatory responses process of microglia activation, which is the vital influence factor of secondary injury after SCI.^16^, ^17^ After necrosis or damage of cells, the NF‐κB signalling pathway is passively released, which activates microglia to secrete a large number of inflammatory cytokines and gradually amplifies the inflammatory response.^10^, ^16^, ^17^ Generally, NF‐κB exists in an inactive form in the cytoplasm through binding with IκBα,^18^ which could be activated by inflammatory factors secreted by microglia with phosphorylation and degradation. Subsequently, the inactive form of NF‐κB transformed to active form and translocated to the cellular nucleus, which regulates the expression of inflammation‐related chemokines and cytokines.^19^, ^20^ A large number of researches have testified that NF‐κB signalling pathway participates in many diseases, such as neuroinflammation,^21^ experimental colitis ^22^ and sepsis.^23^ Hence, regulate NF‐κB signalling pathway could ameliorate the pathology process of these disease. In our study, we explored whether Gramine could regulate NF‐κB signalling pathway involved in promoting motor functional recovery after SCI.

The underlying mechanism of Gramine‐treated microglia may need to be further evaluated, and inhibition of activated microglia by reducing the secretion of inflammatory factors is considered a promising treatment strategy for spinal cord injury. Therefore, our research team tested the possible effect of Gramine on the inflammatory response after SCI in rats and studied the underlying molecular mechanism through LPS‐stimulated microglia.

## 2 MATERIALS AND METHODS

### 2.1 Cell culture and treatment

The microglial cell line (HAPI, CRL‐2815) was obtained from the American Type Culture Collection (ATCC, Manassas, USA). The PC12 cell line was purchased from Cell Biology (Shanghai, China). HAPI and PC12 cells were cultured with high glucose DMEM (Invitrogen, Carlsbad, USA) added 10% foetal bovine serum (FBS, Gibco BRL Co., Ltd., USA), 100 units/ml penicillin and 100 μg/ml streptomycin and incubated at 37℃ in a humidified atmosphere containing 5% CO2 and passaged twice a week. Gramine (Catalog No.S2304, purity ≥98%, Selleckchem, USA) was prepared a stock solution first, which was dissolved in dimethyl sulfoxide (DMSO, Sigma‐Aldrich) to form a 10 mM final concentration. The stock solution of Gramine was diluted appropriately with cell culture medium (final DMSO concentration ≤1‰) for in vitro study. Lipopolysaccharide (LPS, *Escherichia coli* 055:B5, Sigma‐Aldrich) was dissolved in saline and prepared as a stock solution with the final concentration of 5 mg/mL. HAPI cells were pre‐treated with varying dose of Gramine (10, 20, 40, or 80 μM) with or without 1 μg/mL LPS stimulation for 24 hours Gramine was dissolved directly in normal saline before in vivo experiments.

### 2.2 Microglia/neuron co‐culture

To investigate the relationship between microglia and neurons, we utilized a transwell co‐culture system (Corning, 0.4 μm pores, USA). HAPI and PC12 cells were separately seeded into 24‐well (1 × 10^5^ HAPI cells/insert and 2 × 10^5^ PC12 cells/well) transwell plates for the co‐culture experiment. The HAPI cells were pre‐treated Gramine (10 and 20 μM) with or without 1 μg/mL LPS stimulation for 24 hours After stimulation, the HAPI cells were harvested and seeded into inserts being placed into the PC12 wells. After 24 hours of co‐culture, we measured neuronal apoptosis and axonal regeneration by immunofluorescence and Western blot assays.

### 2.3 Cell viability assay

HAPI cells were seeded into a 96‐well plate. After 24 hours for adherence, cells were treated with different dose of Gramine (10, 20, 40 or 80 μM) with or without 1 μg/mL LPS stimulation for 24 hours Then, 10 μL of CCK‐8 (Dojindo, Kumamoto, Japan) solution was added into each well of 96‐well plate and cultured in cell incubator for 4 hours. After incubation, a spectrophotometer (Bio‐Rad, Hercules, USA) was used to detect the OD value at 450 nm.

### 2.4 Nitric oxide assay

Griess reaction was used to measure the Nitrite, which was representative NO production. Briefly, HAPI cells were seeded into a 96‐well plate. After 24 hours for adherence, cells were treated with different dose of Gramine (10 and 20 μM) with or without 1 μg/mL LPS stimulation for 24 hours After incubation, the culture supernatant (100 μL) of each group was added into a 96‐well plate, which was mixed with 100 μL Griess reagent (Beyotime, Shanghai, China) for reacting 10 minutes at room temperature in the dark. After incubation, a spectrophotometer was used to detect the OD value at 540 nm.

### 2.5 ELISA assay

HAPI cells were seeded into a 12‐well plate. After 24 hours for adherence, cells were treated with different dose of Gramine (10 and 20 μM) with or without 1 μg/mL LPS stimulation for 24 hours To detect the anti‐inflammatory effect of Gramine, the cell‐free supernatant of each well was harvested. The concentrations of IL‐1β (MLB00C, R&D Systems, USA), IL‐6 (M6000B, R&D Systems, USA) and TNF‐α (MTA00B, R&D Systems, USA) were detected by the ELISA kits according to the manufacturer's protocols, and OD values were measured by a spectrophotometer at 450 nm.

### 2.6 Real‐time PCR

HAPI cells were re‐plated in 6‐well plates. After 24 hours for adherence, cells were treated with different dose of Gramine (10 and 20 μM) with or without 1 μg/mL LPS stimulation for 24 hours After treatment, TRIzol reagent (15596018, Invitrogen) was added into each well to extract RNA. Then, 1 µL of RNA solution was added into NanoDrop (Thermo Fisher, USA) to detect the concentration and purity of total RNA. 1 μg of total RNA was reverse transcribed into cDNA for the PCR amplification. All cycle threshold values of each gene were harvested and normalized to the β‐actin, a housekeeping gene. In order to find the relative difference in each gene, we used the 2^−ΔΔCt^ method. The primers of all genes used for real‐time PCR are designed by and Sangon Biotech (Shanghai, China) and are shown in Table 1.

**TABLE 1:** Primers used for real‐time PCR analysis

Genes	Forward primers	Reverse primers
IL‐6	GGCGGATCGGATGTTGTGAT	GGACCCCAGACAATCGGTTG
IL‐1β	TGGACCTTCCAGGATGAGGACA	GTTCATCTCGGAGCCTGTAGTG
iNOS	CAGGGAGAACAGTACATGAACAC	TTGGATACACTGCTACAGGGA
TNF‐α	CTCAAGCCCTGGTATGAGCC	GGCTGGGTAGAGAACGGATG
β‐actin	AATGGATTTGGACGCATTGGT	TTTGCACTGGTACGTGTTGAT

### 2.7 Western blotting

Frozen animal spinal cord tissues (0.5 cm in length, at the lesion epicentre) and cells were homogenized in ice‐cold lysis buffer containing 50 mM Tris‐HCl pH 8.0, 150 mM NaCl, 1% NP‐40, 0.5% deoxycholate, 0.1% SDS, 10 mM Na_2_P_2_O_7_, 10 mM NaF, 1 mg/ml aprotinin, 10 mg/ml leupeptin, 1 mM sodium vanadate and 1 mM phenylmethylsulfonyl fluoride (PMSF). Tissue homogenates were incubated for 15 minutes at 4℃ and centrifuged at 12,000 rpm, for 15 minutes at 4℃. The equivalent of 60 μg of total protein was loaded onto SDS‐PAGE and transferred to PVDF membrane (Bio‐Rad). Membranes was blocked by 5% skim milk powder for 2 hours and then incubated with primary antibody at 4℃ for 12 hours Primary antibody including anti‐p‐IκBα (1:500, Cell Signaling Technology, #2859), anti‐IκBα (1:500, Cell Signaling Technology, #4814), anti‐p‐p65 (1:1000, Abcam, ab131100), anti‐p65 (1:1000, Abcam, ab32536), anti‐iNOS (1:1000, Abcam, ab178945), anti‐COX‐2 (1:1000, Abcam, ab179800), anti‐Iba‐1 (1:1000, Abcam, ab48004), anti‐MAP‐2 (1:500, Cell Signaling Technology, #8707), anti‐Bcl‐2 (1:500, Cell Signaling Technology, #3498), anti‐Bax (1:500, Cell Signaling Technology, #89477), anti‐NF‐200 (1:1000, Abcam, ab8135), anti‐CD68 (1:1000, Abcam, ab31630) and anti‐GAPDH (1:500, Cell Signaling Technology, #5174). Then, membranes were incubated with HRP‐conjugated secondary antibody (1:1000, Cell Signaling Technology) for 2 hours in shake cultivation. The membranes were visualized with an electrochemiluminescence plus reagent (Millipore, USA), and images of protein bands were captured on a ChemiDoc XRS^+^ Imaging System (Bio‐Rad, Hercules, USA). All experiments were repeated three times. The intensity of bands was normalized to those of GAPDH using Image Lab 3.0 software.

### 2.8 Molecular modelling

P65 (PDB ID: 2ARM) was chosen for docking studies. Each protein was prepared for docking after being downloaded from PDB (https://www.rcsb.org/). After being minimized using PyMoL (version 1.7.6), the lowest energy conformations for docking were determined via default parameters. The protein‐ligand docking analysis was conducted using AutoDockTools (version 1.5.6), which can provide ligand binding flexibility with the binding pocket residues. The images were finally generated using UCSF PyMoL.

### 2.9 Spinal cord injury model and drug treatment

Total 24 adult female Sprague‐Dawley (SD) rats weighting from 220 to 250 g were obtained from the SLAC Laboratory Animal Company (Shanghai, China) for the in vivo experiments. All the animal procedures were approved by the Institutional Animal Care and Use Committee of Wenzhou Medical University. All the rats were housed under controlled environmental conditions. Before the surgery, animals were deeply anaesthetized by 1% (w/v) pentobarbital sodium (4 mL/kg, i.p.), and a laminectomy was performed at the T9 vertebrae after the vertebral column was exposed. The spinal cord was fully exposed, and a moderate crushing injury was performed using a vascular clip for 1 minutes (30 g forces, Oscar, China). The rats received a single orthotopic injection of Gramine (80 mg/kg, dissolved in saline) or equal volume of saline 30 minutes after the SCI surgery. Postoperative treatments included injection of penicillin solution (4 × 10^6^ units per animal, i.p.) in the first three days and manual bladder emptying twice a day. Subsequently, all rats were deeply re‐anaesthetized and perfused with 0.9% NaCl, followed by 4% paraformaldehyde in 0.01 M phosphate‐buffered saline (PBS, pH = 7.4) at 28 days after surgery.

### 2.10 Locomotion recovery assessment

Locomotion recovery analyses, including the Basso‐Beattie‐Bresnahan (BBB) locomotion scale^24^ and the inclined plane test, were performed at 0, 1, 3, 7, 14, 21 and 28 days, and footprint analysis ^25^ was performed at 28 days. Rats were placed in an open experimental field and allowed to move freely for 5 minutes. Crawling ability was assessed by the BBB scale ranging from 0 (no limb movement or weight support) to 21 (normal locomotion), and the footprint analysis was performed by dipping the animal's posterior limb with red dye and forelimb with blue dye.^26^ The inclined plane test was also performed to assess functional improvement in each rat at each of the above time points. Outcome measures were obtained by 3 independent examiners who were blinded to the experimental conditions.

### 2.11 Tissue preparation

Animals were anaesthetized by 1% (w/v) pentobarbital sodium (4 mL/kg, i.p.) at specific time points following SCI. For immunofluorescence staining, 0.5 cm section of the spinal cord was dissected out, post‐fixed by 4% paraformaldehyde for 6 hours and then embedded in paraffin. Longitudinal sections (5 µm thick) were mounted on slides for subsequent staining.

### 2.12 Haematoxylin and eosin staining

The longitudinal sections were dewaxed and washing with PBS. Then, sections were fixed with acetone for 2 minutes, washed with water for 1‐2 s and stained with haematoxylin for 5 minutes at room temperature. After washing, the slides were immersed in 1% acidic alcohol for 5‐10 s and rinsed twice (2 minutes per rinse) with distilled water. Eosin solution was added to re‐stain the sections for 1‐2 minutes, followed by washing and dehydrating through a gradient ethanol series (80%, 90% and 100%; 30 s per step). Finally, the sections were immersed in xylene I (5 minutes) and xylene II (5 minutes) and sealed with neutral gum. The slides were observed, and images were acquired under a light microscopy (Olympus, Tokyo, Japan).

### 2.13 Luxol fast blue staining

The longitudinal sections were dewaxed followed by washed with PBS. The sections were immersed in 0.1% LFB dye at room temperature overnight, washed and de‐stained with 0.05% lithium carbonate solution for 5 minutes, followed by dehydration with a graded series of ethanol (70% 10 s, 95% 25 s, 100% 25 s). Finally, the slides were hyalinized with xylene and mounted with neutral gum. The images were also acquired under a light microscopy (Olympus, Tokyo, Japan).

### 2.14 Immunofluorescence

In vitro: HAPI cells were seeded into 6‐well plates. After 24 hours for adherence, cells were treated with different dose of Gramine (10 and 20 μM) with or without 1 μg/mL LPS stimulation for 24 hours Co‐cultured PC12 cells were seeded into 24‐well plates. After adherence, washed twice by PBS. Then, 1 mL 4% paraformaldehyde was added into each well for 30 minutes. Then, 1 mL 0.5% Triton X‐100 was added into each well for permeabilization 15 minutes, followed by blocking with 5% BSA for 1 hours at room temperature. HAPI cells were incubated with primary antibody: anti‐p65 (1:400, Abcam) or anti‐Iba‐1 (1:500, Abcam) at 4℃ for 12 hours PC12 cells were incubated with primary antibody: anti‐MAP‐2 (1:400, Cell Signaling Technology) or anti‐Bax (1:500, Cell Signaling Technology) at 4℃ for 12 hours After incubation, Dylight 488 and Dylight 550 donkey anti‐rabbit/goat secondary antibodies were added for culture 1 hours at 37℃ in the dark. After incubation, cell nucleus was stained with DAPI (Beyotime). The HAPI and PC12 cells were visualized by a fluorescence microscope (Leica, Germany). Finally, three fields of view were selected randomly in each group of cells and the fluorescence intensity or positive cell number were measured by Image‐Pro Plus.

In vivo: The longitudinal sections were performed with antigen retrieval in 0.01 M citrate buffer solution (pH = 6.0) after deparaffinization. Then, sections were treated with primary antibodies targeting the following proteins: NF‐200 (1:2000, Abcam, ab8135), GFAP (1:2000, Abcam, ab53554), MAP‐2 (1:300, Cell Signaling Technologies, #8707) and CD68 (1:400, Abcam, ab31630). The slides were washed four times with PBST and incubated with Dylight 488 and Dylight 550 donkey anti‐rabbit/mouse/goat secondary antibodies for 1 hours at 37℃. Afterwards, the sections were washed with PBS, incubated with DAPI for 7 minutes, rinsed with PBS and finally sealed with a coverslip. All the images were captured by a fluorescence microscope (Leica, Wetzlar, Germany). The results of immunofluorescence staining were quantified by mean fluorescence density with ImageJ software 1.52V (National Institutes of Health, Bethesda, MD, USA).

### 2.15 Statistical analysis

The results were presented as mean ± SD Statistical analyses were performed using SPSS statistical software program 18.0, which were from three independent experiments. Difference among groups was assessed by the one‐way analysis of variance (ANOVA) followed by Tukey test. *P value <0.05 was considered as statistically significant.

## 3 RESULTS

### 3.1 Gramine suppresses microglia secreting pro‐inflammatory cytokines and expressing inflammatory‐related genes

**FIGURE 1:**
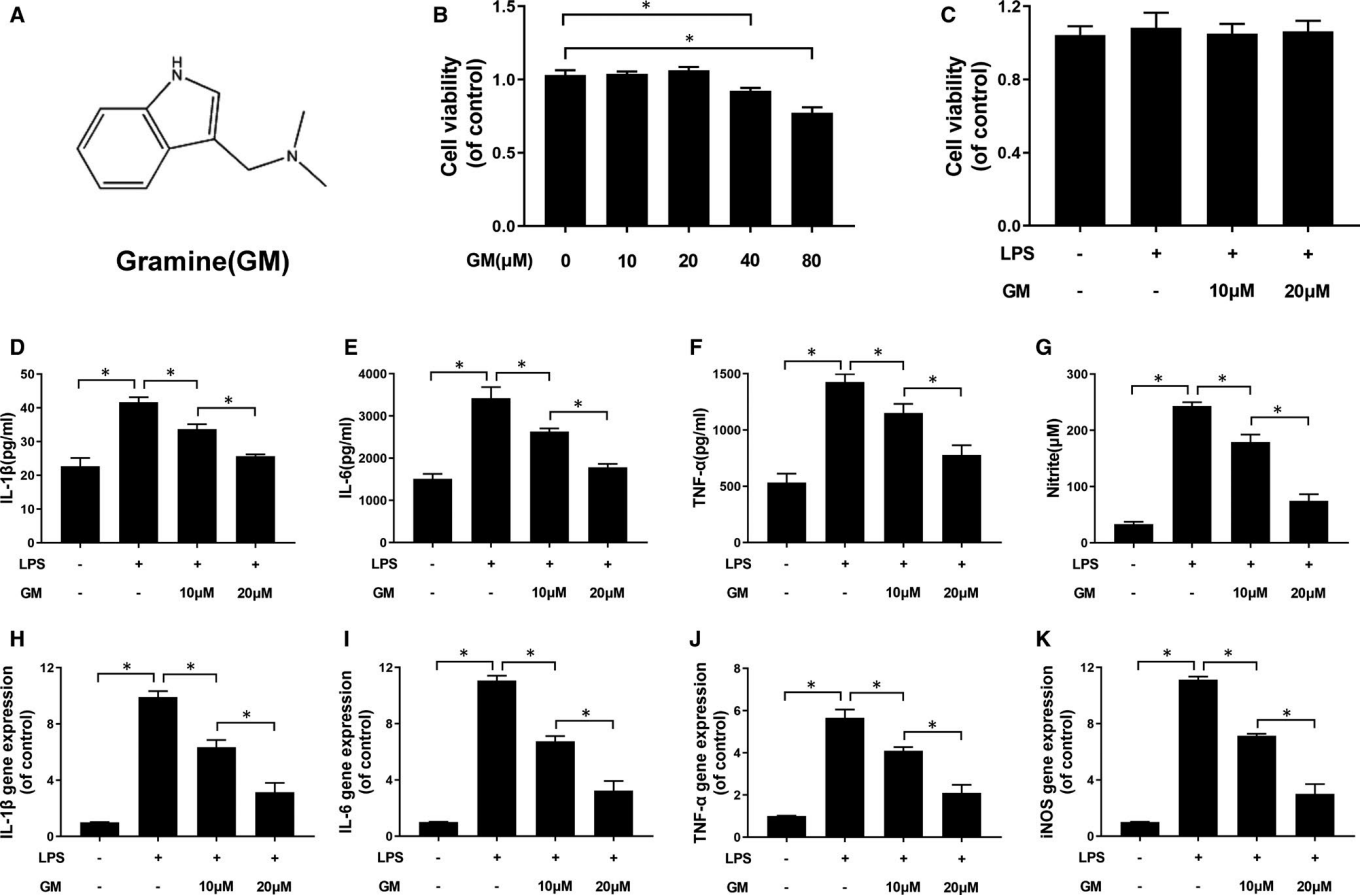
Gramine inhibited the release of pro‐inflammatory mediators in microglia. A, The chemical structure of Gramine. (B, C) The cell viability of microglia treated with Gramine at different concentrations with or without LPS for 24 hours and was evaluated by CCK‐8 assay. D‐G, The levels of pro‐inflammatory mediators after Gramine treatment in microglia. H‐K, The result of inflammation‐related genes expression levels in microglia. The values presented are the means ± SD *P < 0.05 versus control group, n = 3

The chemical structure of Gramine has been exhibited in Figure 1A. To evaluate cytotoxicity of Gramine, HAPI cells were treated with Gramine different concentrations of Gramine. CCK‐8, which was used to measure the cell viability, and revealed that Gramine caused no significant cytotoxicity on HAPI cells at concentrations of ≤20 μM (Figure 1B). However, microglia after 40 and 80 μM Gramine treatment showed viability decreased to 92.45% ± 0.02 and 77.45% ± 0.04, respectively. Moreover, the viability of microglia pre‐treated with 0‐20 μM Gramine followed by LPS (1 μg/mL) treatment was no statistical significance compared with control group (Figure 1C). Therefore, 10 and 20 μM Gramine were chose for our further study. We evaluated the effect of Gramine on the pro‐inflammatory mediator release in HAPI cells in vitro and detected the pro‐inflammatory cytokines, such as IL‐1β, IL‐6, TNF‐α and NO secreted from LPS‐induced microglia. As shown in Figure 1D‐F, IL‐1β, IL‐6 and TNF‐α those pro‐inflammatory cytokines were significantly increased after LPS stimulation, whereas remarkably decreased in dose‐dependent manner by Gramine treatment. Moreover, we detected the extent of NO, which was synthesized by the critical enzyme, inducible nitric oxide synthase (iNOS) of inflammatory response. Our group detected a remarkable increase of NO release by LPS stimulation, whereas it reversed after pre‐treatment with Gramine (Figure 1G). Furthermore, inflammation‐related mRNAs were evaluated by qPCR. Data demonstrated that IL‐1β, IL‐6, TNF‐α and iNOS were remarkably increased by LPS‐stimulated and reversed after Gramine pre‐treatment (Figure 1H‐K). These data demonstrated that LPS improved the higher expression of pro‐inflammatory mediators in microglia, but Gramine pre‐treatment can significantly attenuate its increase.

### 3.2 Gramine inhibits LPS‐induced microglia activation

**FIGURE 2:**
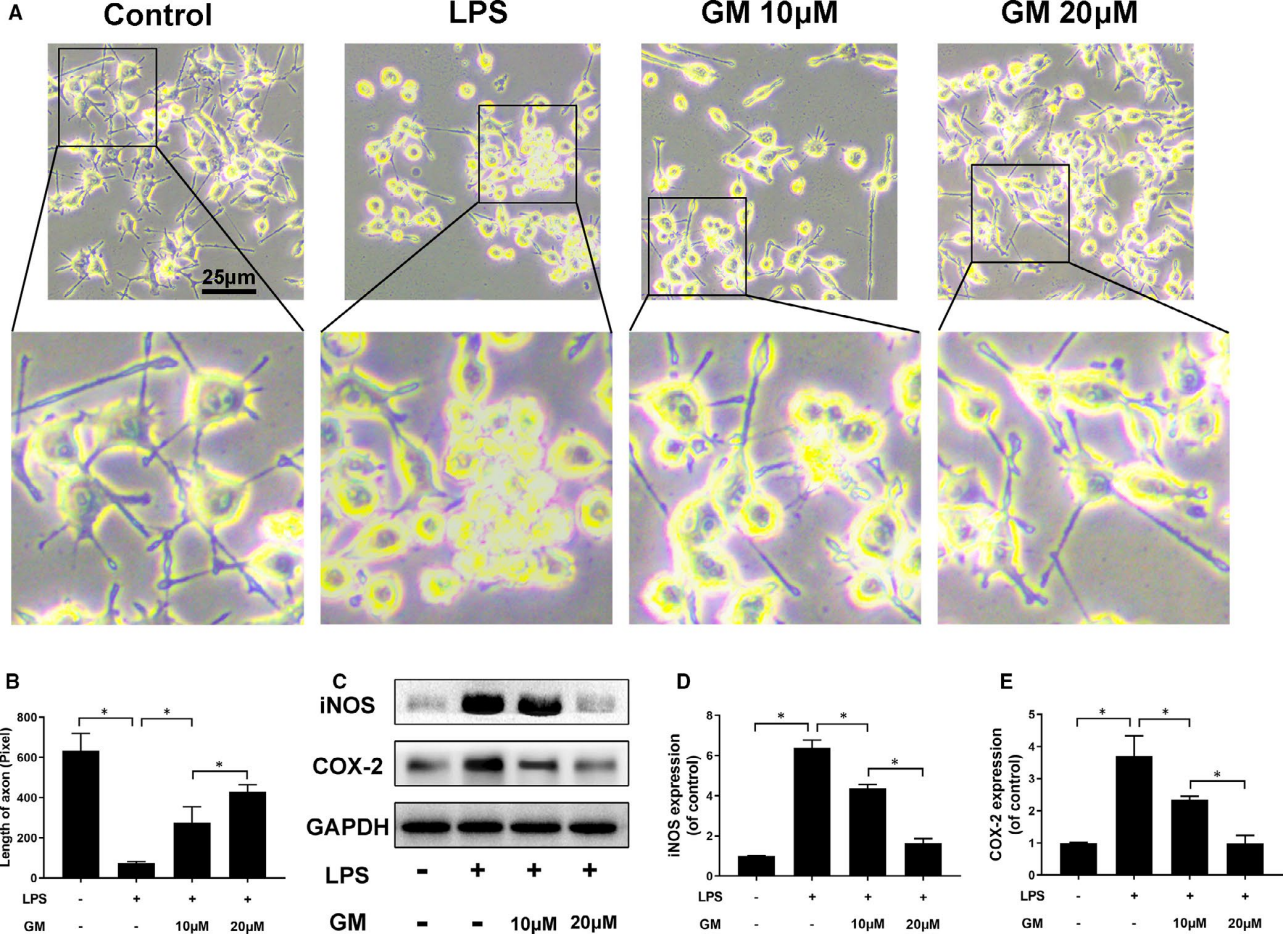
Gramine suppressed LPS‐induced microglial cells activation. A, The morphological results of microglia. B, Quantification analysis of morphological changes by measuring the length of axonal. C‐E, Western blot results and quantification data of iNOS and COX‐2 in microglia. The values presented are the means ± SD, *P < 0.05 *vs*. indicated group, n = 3

Microglia exhibit branching morphology under normal condition, whereas the branch axon would retract showing an amoeboid shape after LPS, IFN‐γ and β‐amyloid stimulation.^9^, ^27^ Similarly, our results show that HAPI microglia cells retract the axon to form an amoeboid shape after LPS stimulation, whereas the morphology would be reversed by Gramine pre‐treatment (Figure 2A). As shown in Figure 2B, we measured the axonal length of microglia in different condition, showing that the length decreased about at 75.25 ± 7.27 pixel followed by LPS stimulation compared with control group (634.5 ± 85.5 pixel) and restored 275.5 ± 79.6 and 428.5 ± 35.9 pixel by Gramine pre‐treatment in a dose‐dependent manner, respectively. Moreover, we evaluated the protein expression of iNOS and COX‐2, which were hallmarks of inflammatory reaction of microglia. Our results demonstrated that iNOS and COX‐2 were significantly enhanced by LPS stimulation and restored followed by Gramine pre‐treatment (Figure 2C‐E).

### 3.3 Gramine inhibits activation of microglia and promotes axonal regeneration of neuron

**FIGURE 3:**
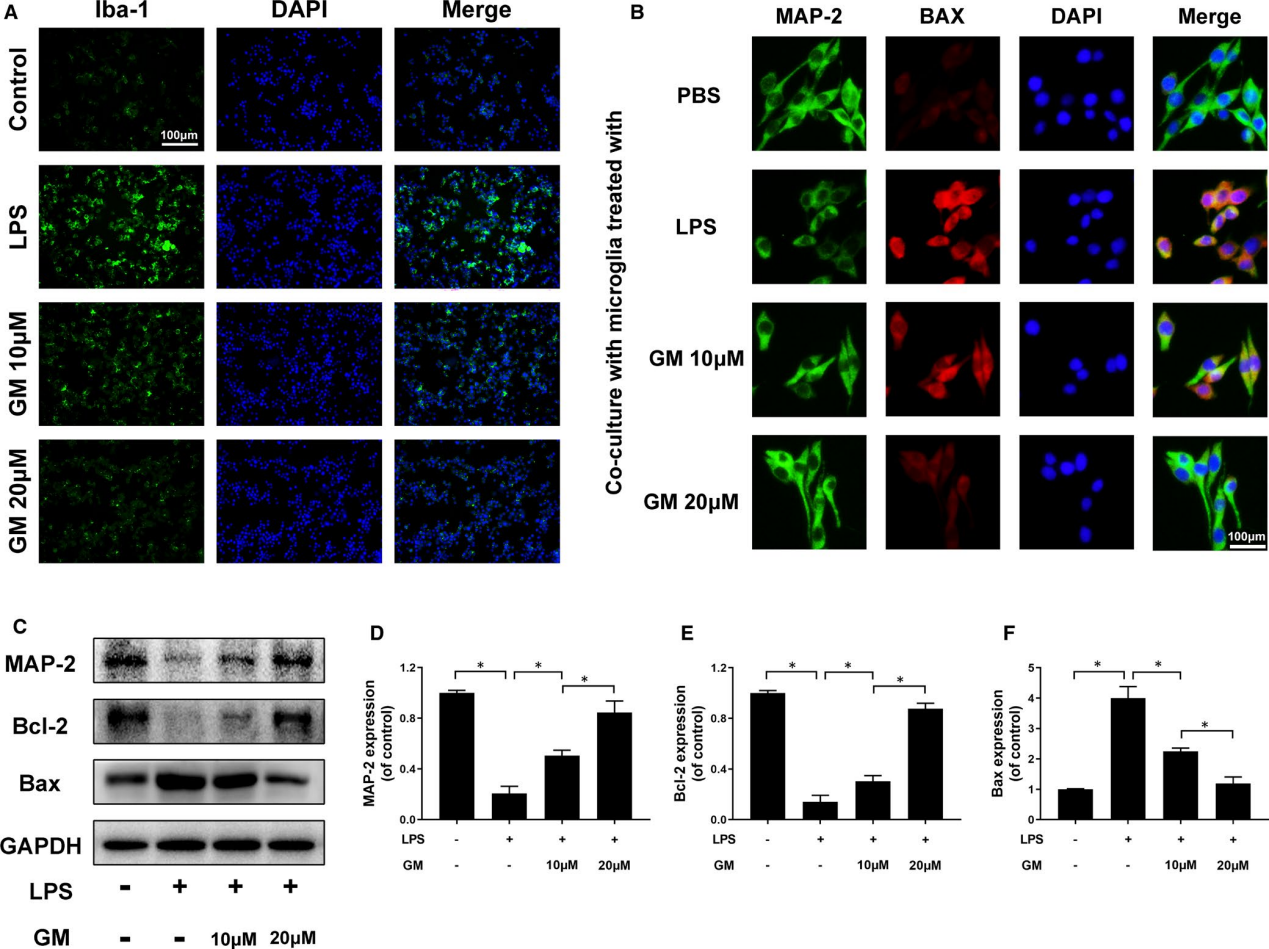
Gramine suppresses microglia activation and promote neuron survival. A, Immunofluorescence staining for Iba‐1 in microglia. B, Double immunofluorescence staining results of MAP‐2 and Bax by neurons co‐cultured with Gramine pre‐treated microglia. C‐F, Western blot and quantification analysis of MAP‐2, Bcl‐2 and Bax. The values presented are the means ± SD *P < 0.05 versus indicated group, n = 3

In our study, we detected the Iba‐1, which is a hallmark of activated microglia by immunofluorescence to evaluate the anti‐inflammatory effect of Gramine. Our data show that the fluorescence intensity of Iba‐1 was remarkably increased after LPS stimulation, whereas reversed by Gramine pre‐treatment in a dose‐dependent manner (Figure 3A). Moreover, a co‐culture system was used to assess the effects of activated microglia on neurons. We treated the microglia firstly using different dose of Gramine (10 and 20 μM). After that, treated microglia were harvested and added into a co‐culture system, which worked with neurons. We used immunofluorescence staining to assess the apoptosis and axonal regeneration of neuron after co‐culture with microglia.

As shown in Figure 3B, neurons performed axonal regeneration at a remarkably decreased rate (green fluorescence intensity) and apoptosis (red fluorescence intensity) at a significantly increased rate co‐cultured with activated microglia. Interestingly, the axonal regeneration and apoptosis rate of neurons were restored by co‐cultured with Gramine pre‐treatment microglia. Furthermore, axon‐related proteins (MAP‐2) and apoptosis‐related proteins (Bcl‐2 and Bax) were measured by Western blotting, showing the axonal regeneration associated with apoptosis (Figure 3C‐F).

### 3.4 Gramine suppresses microglia activation via NF‐κB signal pathway

**FIGURE 4:**
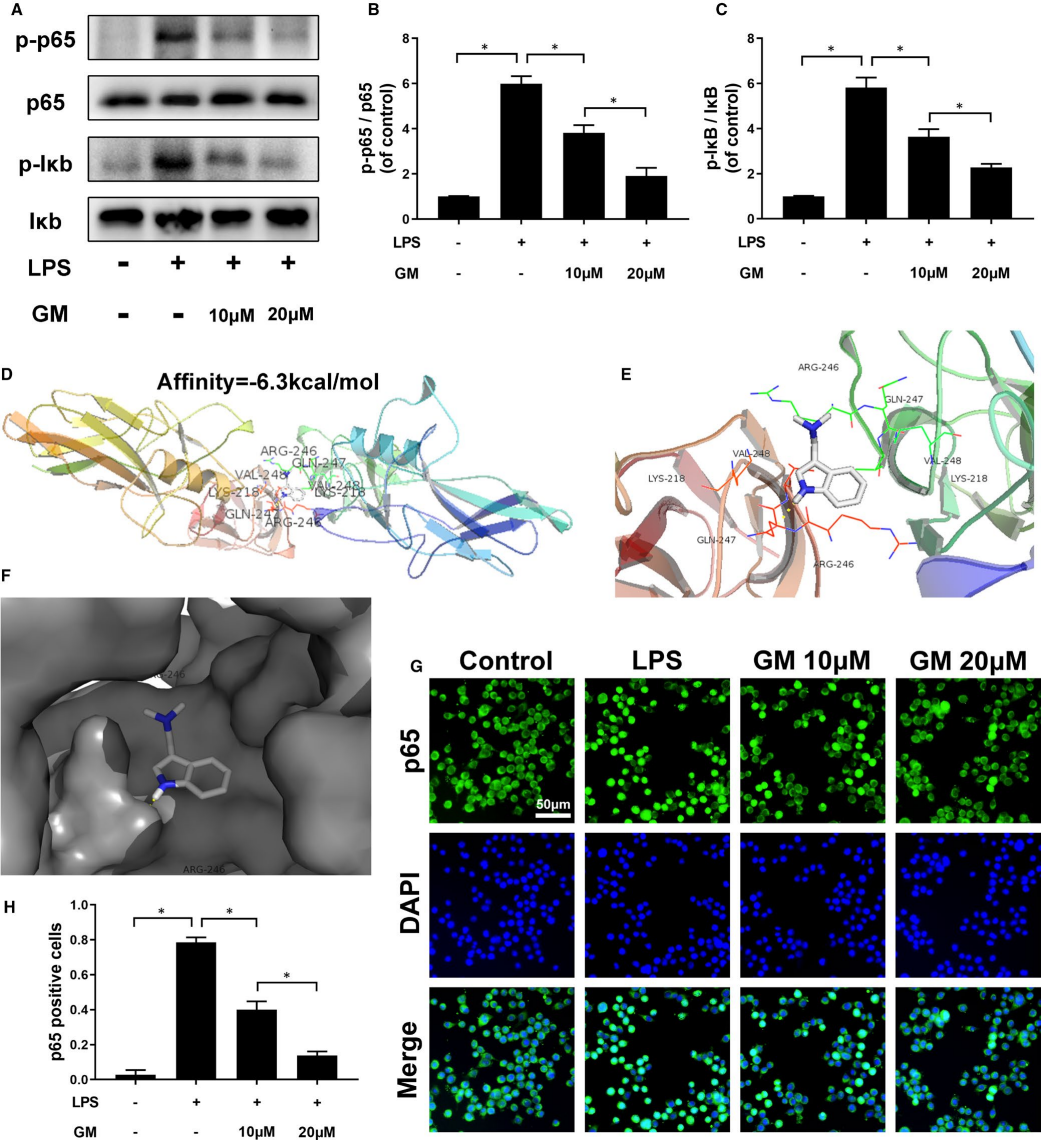
Gramine regulates activation of the NF‐κB pathway. A‐C, Western blot and quantification analysis of phosphorylated IκB and NF‐κB in microglia. D‐F, The protein residues are shown in a ribbon model. The space‐filling models show the binding of Gramine in the inhibitory binding pockets. G, H, Immunofluorescence staining and quantification results of NF‐κB in microglia. The values presented are the means ± SD, *P < 0.05 versus indicated group, n = 3

NF‐κB and IκBα were critical proteins in NF‐κB signal pathway; the Western blot results show that these proteins were phosphorylated after LPS stimulation and dephosphorylated followed by Gramine pre‐treatment in a dose‐dependent manner (Figure 4A‐C). In order to investigate the affinity between NF‐κB and Gramine, we used a molecular docking analysis according to diverse binding pockets of the antagonist. By studying all the models returned, we found that Gramine formed some favourable connections and docked nicely within the NF‐κB binding sites (Figure 4D). In Figure 4D and E, the macro views and the views of the local interactions of protein residues are shown in a ribbon model. Meanwhile, space‐filling models (Figure 4F) are used to directly illustrate the coverage of Gramine in the related protein structures. According to the docking with the NF‐κB structure (Figure 4D and E), some important hydrogen bonds were formed between Gramine and NF‐κB concerning GLN‐247, ARG‐246 and LYS‐218, with a high affinity of −6.3 kcal mol^−1^. Furthermore, the nuclear translocation of NF‐κB was detected by immunofluorescent showing that NF‐κB protein would translocate to the nucleus after LPS stimulation which means the activation of microglia. Moreover, this phenomenon could be suppressed by Gramine pre‐treatment, NF‐κB protein performed at cytoplasm mostly (Figure 4G and H).

### 3.5 Gramine exerts neuroprotective effects on spinal cord injury

**FIGURE 5:**
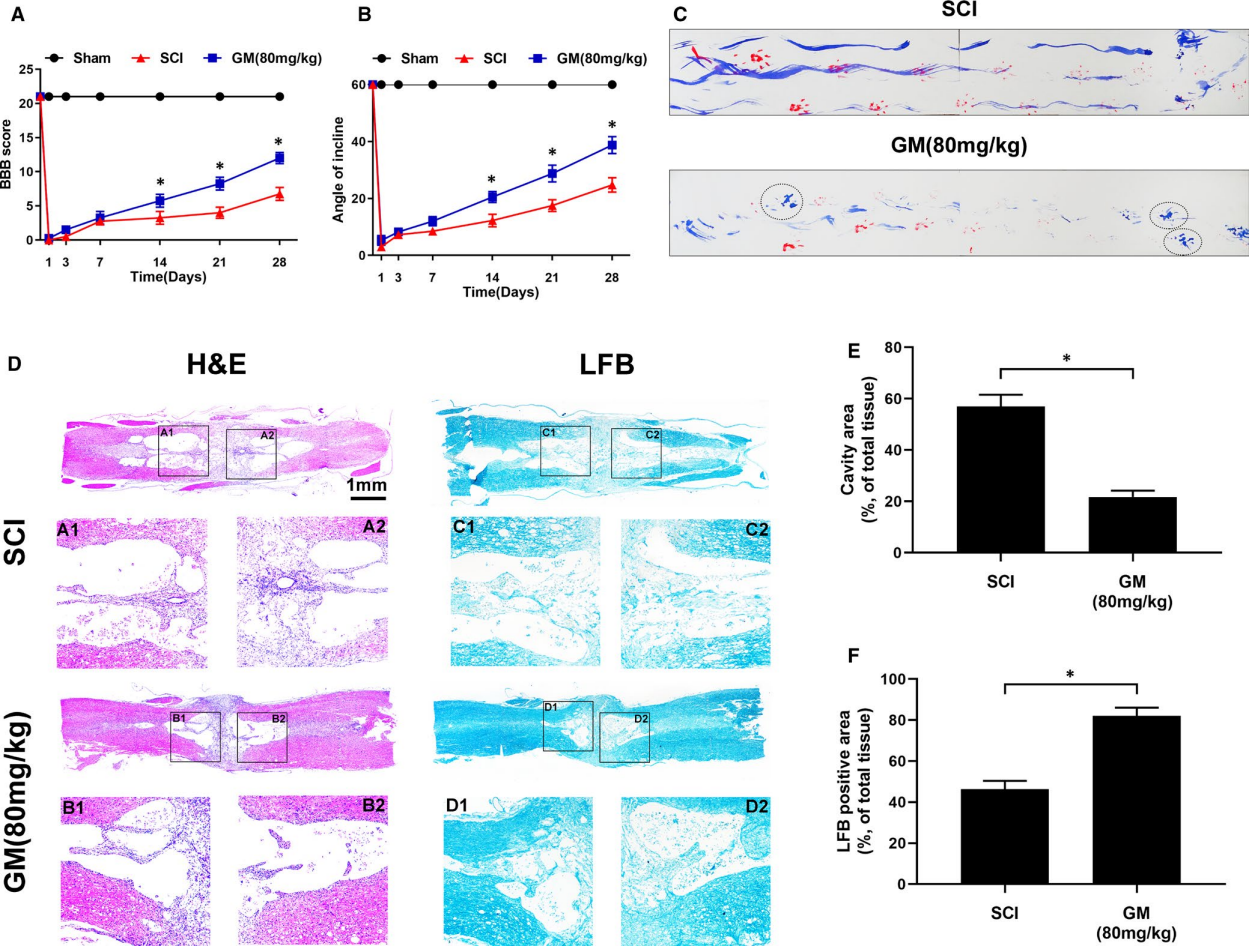
Gramine improved pathology and motor function after SCI. A, The Basso, Beattie and Bresnahan (BBB) limb function scores at different times after spinal cord injury. B, Inclined plane test scores at different times after spinal cord injury. C, Representative footprints of an animal walking 28 days after SCI. Red: forelimb print; blue: posterior limb print. The dotted circles show the fairly consistent posterior limb. D, H&E and LFB stains at 14 days. E, Graphic presentation of the percentage of cavity area of the spinal cord. F, Quantification data of LFB‐positive area in spinal cord. The values presented are the means ± SD, *P < 0.05 versus indicated group, n = 3

Behavioural experiments including BBB scores, the inclined plane test and footprint analysis were measured to assess the functional recovery of SCI.^1^ Interestingly, our results show that Gramine treatment group could achieve a nice BBB scores compared with SCI group. Moreover, the BBB scores of SCI group could also tardily return to Gramine treatment group (Figure 5A). Similarly, the inclined plane test also showed the excellent behaviour with the higher angles of Gramine treatment group compared with SCI group (38.75 ± 2.99 vs 24.75 ± 2.5) (Figure 5B). In footprint analysis, Gramine‐treated rats showed a fairly consistent posterior limb (blue ink) coordination and a few stumbled walking tracks, which were pointed out by dotted circles. On the contrary, SCI group animal showed inconsistent behaviour, and with extensive dragging, as determined by the presence of ink streaks extending from both posterior limbs (Figure 5C). Haematoxylin‐eosin staining was used to evaluate the morphological change, which shows the cavity area of Gramine treatment group was significantly smaller than SCI group (Figure 5D and E). Luxol fast blue staining was performed to assess the extent of remyelination after SCI, which shows the myelin sheath destruction was suppressed and the LFB‐positive myelin was remarkably enhanced by Gramine treatment (Figure 5D and F).

### 3.6 Gramine suppresses microglia/macrophage activation after SCI

**FIGURE 6:**
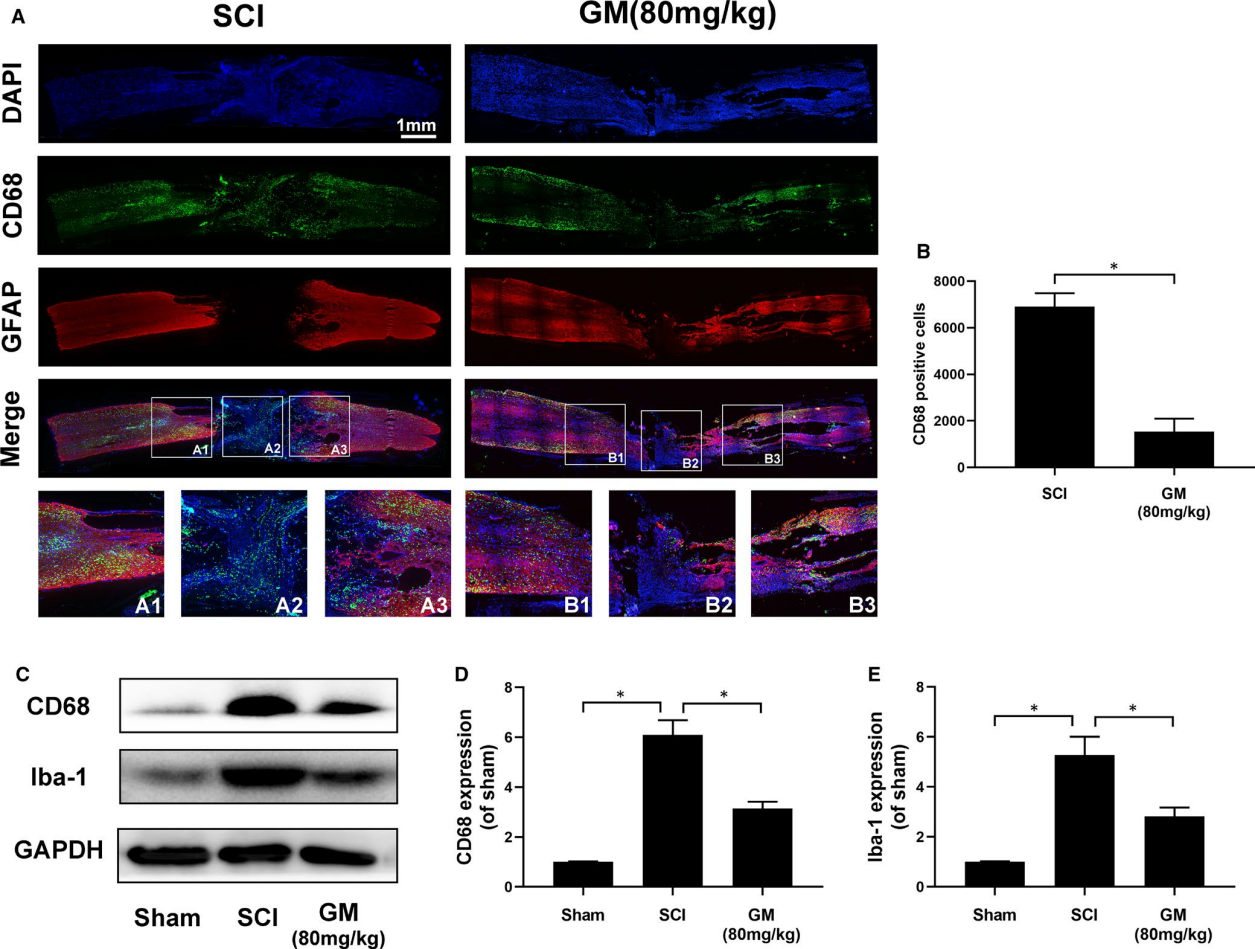
Gramine suppresses microglia activation after SCI. A, Double‐fluorescence staining analysis for CD68 (green)/GFAP (red) of spinal cord tissue sections. B, Graphic presentation of the CD68‐positive cells in injured spinal cord. C‐E, Western blot and quantification analysis of CD68 and Iba‐1. The values presented are the means ± SD, *P < 0.05 vs. indicated group, n = 3

As shown in Figure 6A, CD68 (Green) and GFAP (Red) were double‐dyed to watch the number and distribution of activated microglia/macrophage. Compared with the SCI group, the distribution of positive expression cells (CD68) in the Gramine treatment group significantly reduced (Figure 1, 6).

Western blot analysis of CD68 and Iba‐1 also confirmed the above results, showing that CD68 and Iba‐1 proteins expression were prominently decreased after Gramine treatment (Figure 6C‐E) which suggested Gramine could reduce the inflammatory response after SCI in rats.

### 3.7 Gramine improved axonal regeneration after SCI

**FIGURE 7:**
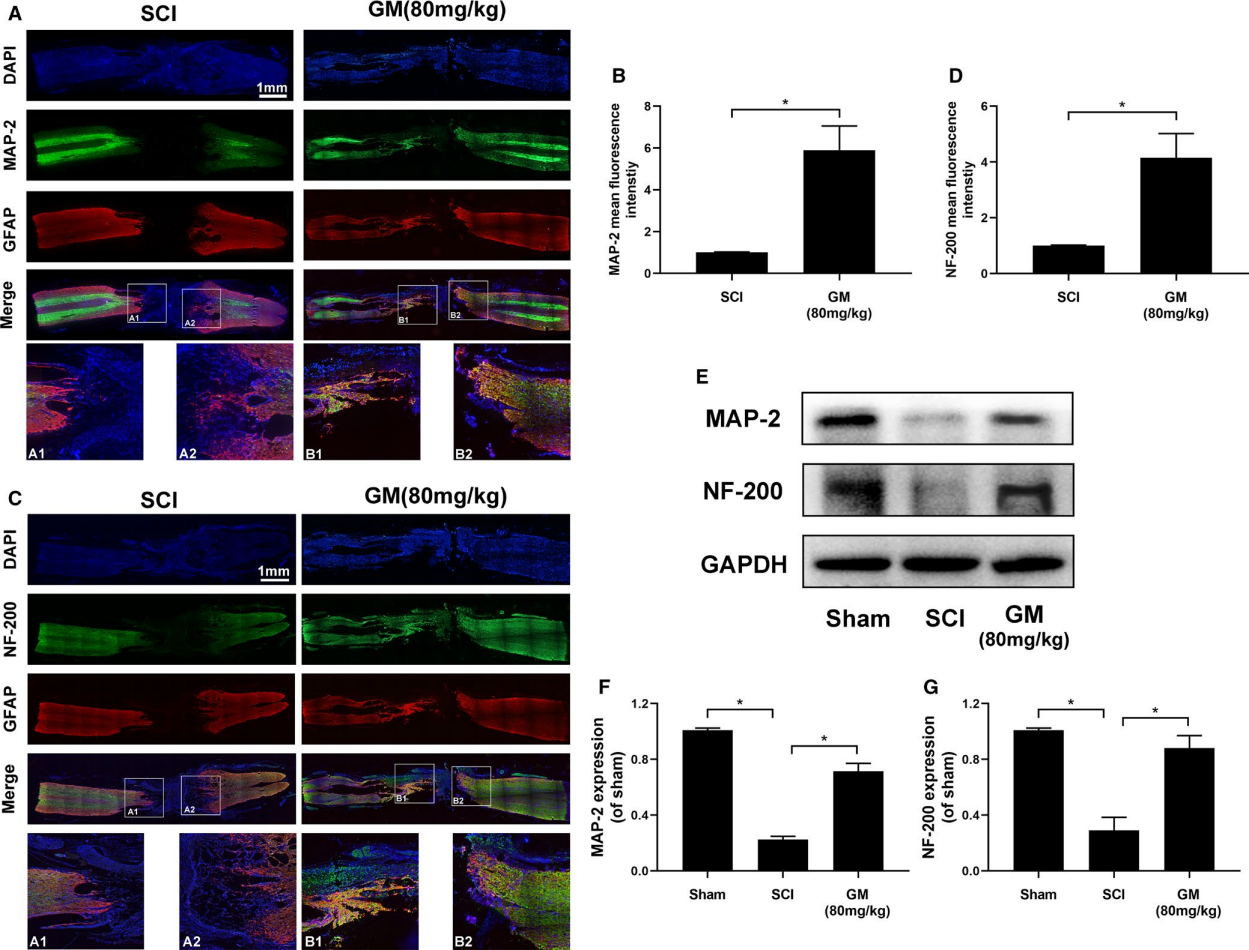
Gramine promotes neural repair and axonal regeneration after SCI. A, Double immunofluorescence analysis showed MAP‐2 (green) and GFAP (red) in each group. B, Graphic presentation of the MAP‐2 mean fluorescence. C, Double immunofluorescence analysis showed NF‐200 (green) and GFAP (red) in each group. D, Graphic presentation of the NF‐200 mean fluorescence. E‐G, Western blot and quantification analysis of MAP‐2 and NF‐200. The values presented are the means ± SD, *P < 0.05 versus indicated group, n = 3

Co‐immunostaining was performed with anti‐MAP‐2 antibody to label neurons and anti‐GFAP antibody to label astrocyte. As shown in Figure 7A, GFAP‐positive astrocytes, which named glial scar, were distributed along the lesion border. Notably, Gramine treatment group showed a narrower lesion area compared to SCI group. Furthermore, in Gramine treatment group, MAP‐2‐positive neurons remarkably increased compared with SCI group in the lesion border (Figure 7A). In addition, we measured the fluorescence intensity of MAP‐2, suggesting that the animals in Gramine treatment group significantly prevented robust loss of neurons (Figure 7B). From the perspective of motor functional recovery, axon regeneration is vital. Therefore, the extension of neurofilaments was evaluated by NF‐200 and GFAP double‐immunostaining (Figure 7C and D), showing the number of NF‐200‐positive neurofilaments were significantly boost and these neurofilaments could traverse the glial scar and present extended axons by Gramine treatment. In addition, Western blot showed that Gramine treatment greatly enhanced the expression of MAP‐2 and NF‐200 (Figure 7E‐G) than the SCI group, showing a similar trend to immunofluorescence. It is suggested that the treatment of Gramine is helpful for the repair of nerves and axons after SCI, thereby promoting the functional recovery after SCI.

## 4 DISCUSSION

SCI is a serious disabling disease, and its incidence is about 12,000 new cases every year. The main feature is the obvious loss of sensory and motor functions.^28^ Therefore, it is imperative to find effective strategies for treating SCI. At present, increasingly studies have proved that Chinese herbal medicine, especially its bioactive compounds can also produce obvious therapeutic effects on spinal cord injury.^1^, ^29^, ^30^, ^31^ Recently, extraction of herb performed nice neuroprotection effects in central nervous system, such as Parkinson's disease,^27^ Alzheimer's disease ^32^ and SCI.^33^ Gramine, an indole alkaloid, exhibits various bioactive properties. Recently, Ramu et al ^15^ demonstrated that Gramine plays an anti‐proliferative and anti‐inflammatory agonist effect on the occurrence of oral cancer carcinogenesis; however, the underlying mechanism of Gramine in neuroprotective effects and its anti‐inflammatory effects in microglia are still unclear. In this study, we evaluated the effect of Gramine in SCI and explored the molecular mechanism of its neuroprotective effects. The behavioural experiments results indicated that SCI rats would achieve a remarkable recovery of motor function after Gamine treatment. Moreover, histology staining demonstrated that Gramine prevented the cavity formation and promoted myelin sheath restoration after spinal cord injury. In addition, CD68‐positive cells were detected by immunofluorescence staining, which show that the number of CD68‐positive cells in the injured site of spinal cord was noteworthily decreased after Gramine treatment.

Furthermore, more MAP‐2‐positive neurons and NF‐200‐positive neurofilament were detected at the damaged edge and centre of spinal cord after Gramine treatment. Additionally, Western blot assay confirmed that MAP‐2 and NF‐200 expression were consistent with immunofluorescence staining. Those results indicate that Gramine may improve axonal regeneration and remyelination may through suppressing microglia activation after SCI. Followed by the primary injury, secondary injury, microglia or macrophage‐mediated inflammatory reaction exacerbates spinal cord tissue damage and functional loss.^5^, ^33^, ^34^ Activated microglia or macrophages secreted numerous pro‐inflammatory cytokines and cytotoxic mediators, which caused demyelination and neuronal loss in central nervous system.^35^, ^36^, ^37^ Therefore, our group want to explore the underlying mechanism of Gramine in central nervous system for its neuroprotection effects.

Inducible nitric oxide synthase (iNOS) could be induced when microglia or macrophages are activated after spinal cord injury. It could synthase nitric oxide (NO), which has a neurotoxic effect in central nervous system.^38^, ^39^, ^40^ Moreover, iNOS could be noteworthily boosted by other inflammation‐related mediators via a positive feedback cascade.^41^, ^42^, ^43^ In our experiments, the production of NO was suppressed and the key enzyme of NO synthesis, iNOS also be inhibited. Our study demonstrates that LPS‐activated microglia significantly upregulated the expression of iNOS TNF‐α, IL‐1β and IL‐6. Furthermore, the downstream target proteins of these proteins, such as NO, are activated accordingly,^44^ thereby enhancing the inflammatory response associated with acute and chronic inflammation. In our study, when induced by LPS, both iNOS and COX‐2 were significantly upregulated, both in Western blot and qPCR results, consistent with previous study results. However, their expression levels were lower after Gramine treatment. That proves that Gramine reduces neuroinflammatory damage by downregulating pro‐inflammatory factors. A mass of studies has reported that the activation of microglia play a vital role in central nervous system diseases,^45^, ^46^ which has verified by our results that Iba‐1 expression was decreased after Gramine treatment. Furthermore, we used co‐culture system to verify the neuroprotective effect of Gramine. The results showed that axonal regeneration of PC12 cells was remarkably increased and apoptosis of PC12 cells was significantly decreased after co‐cultured with Gramine pre‐treatment microglia. Thus, Gramine is identified as an anti‐inflammatory medicine on microglia, which could promote axonal regeneration of neurons; however, the underlying mechanism of the anti‐inflammation effects of Gramine is still unclear.

There are numerous pro‐inflammatory mediators involved in initiating inflammation, and NF‐κB is a key protein, which could regulate the expression of iNOS and COX‐2.^47^, ^48^ The complex formed by NF‐κB and IκB‐α resides in the cytoplasm of resting cells. When the NF‐κB signalling pathway is stimulated and activated by extracellular factors such as LPS and IL‐1β, the complex formed by IκB‐α and NF‐κB will be separated into monomers and phosphorylated, respectively. Finally, phosphorylated NF‐κB will translocate to the nucleus. In our study, a docking analysis demonstrated that NF‐κB has an active binding domain for attracting Gramine. Consistent with our Western blot and immunofluorescence results that the expression of phosphorylated NF‐κB and IκB‐α was decreased after Gramine treatment. It suggested that Gramine could combine with NF‐κB and then perform suppressing of NF‐κB activation and nuclear translocation.

## 5 CONCLUSION

To sum up, our research shows that Gramine inhibits the high expression of LPS‐induced pro‐inflammatory mediators and neuronal apoptosis by regulating NF‐κB signalling pathways. We observed in both that Gramine can exert neuroprotective effects by inhibiting excessive activation of microglia in vivo and in vitro. The above research proves that Gramine may become a potential drug for SCI therapy.

## CONFLICT OF INTEREST

The authors confirm that there are no conflicts of interest.

## AUTHOR CONTRIBUTIONS

**Xiaolang Lu:** Data curation (lead); Investigation (equal); Methodology (equal); Software (equal); Validation (equal); Visualization (equal); Writing‐original draft (lead); Writing‐review & editing (equal). **Fengfeng Lu:** Formal analysis (lead); Methodology (equal); Resources (equal); Software (equal); Supervision (equal); Validation (equal). **Jiachen Yu:** Methodology (supporting); Software (supporting). **xinghe Xue:** Data curation (lead); Investigation (equal); Methodology (equal); Software (equal); Validation (equal); Visualization (equal); Writing‐original draft (lead); Writing‐review & editing (equal). **Hongyi Jiang:** Investigation (equal); Methodology (equal); Software (equal); Validation (equal); Visualization (equal); Writing‐original draft (equal). **Liting Jiang:** Data curation (equal); Investigation (equal); Methodology (equal); Software (equal); Visualization (equal). **Yang Yang:** Conceptualization (lead); Data curation (equal); Funding acquisition (equal); Project administration (equal).

## Data Availability

The data sets generated and/or analysed during the current study are not publicly available but are available from the corresponding author on reasonable request.
